# Ankyrin B and Ankyrin B variants differentially modulate intracellular and surface Cav2.1 levels

**DOI:** 10.1186/s13041-019-0494-8

**Published:** 2019-09-02

**Authors:** Catherine S. W. Choi, Ivana A. Souza, Juan C. Sanchez-Arias, Gerald W. Zamponi, Laura T. Arbour, Leigh Anne Swayne

**Affiliations:** 1 Division of Medical Sciences, University of Victoria, Victoria, British Columbia Canada; 2 Department of Physiology and Pharmacology, Hotchkiss Brain Institute and Alberta Children’s Hospital Research Institute, Cumming School of Medicine, University of Calgary, Calgary, Alberta Canada; 3 Department of Medical Genetics, University of British Columbia, Vancouver, British Columbia Canada

**Keywords:** Ankyrin B, Cav2.1, CACNA1A, Intracellular pool, Surface localization, Synapse

## Abstract

Ankyrin B (AnkB) is an adaptor and scaffold for motor proteins and various ion channels that is ubiquitously expressed, including in the brain. AnkB has been associated with neurological disorders such as epilepsy and autism spectrum disorder, but understanding of the underlying mechanisms is limited. Cav2.1, the pore-forming subunit of P/Q type voltage gated calcium channels, is a known interactor of AnkB and plays a crucial role in neuronal function. Here we report that wildtype AnkB increased overall Cav2.1 levels without impacting surface Cav2.1 levels in HEK293T cells. An AnkB variant, p.S646F, which we recently discovered to be associated with seizures, further increased overall Cav2.1 levels, again with no impact on surface Cav2.1 levels. AnkB p.Q879R, on the other hand, increased surface Cav2.1 levels in the presence of accessory subunits α_2_δ_1_ and β_4._ Additionally, AnkB p.E1458G decreased surface Cav2.1 irrespective of the presence of accessory subunits. In addition, we found that partial deletion of AnkB in cortex resulted in a decrease in overall Cav2.1 levels, with no change to the levels of Cav2.1 detected in synaptosome fractions. Our work suggests that depending on the particular variant, AnkB regulates intracellular and surface Cav2.1. Notably, expression of the AnkB variant associated with seizure (AnkB p.S646F) caused further increase in intracellular Cav2.1 levels above that of even wildtype AnkB. These novel findings have important implications for understanding the role of AnkB and Cav2.1 in the regulation of neuronal function in health and disease.

## Introduction

Ankyrin B (AnkB) is a scaffolding adaptor protein that plays a variety of roles in multiple systems including the brain, heart, and pancreas [1–6]. In neurons, it acts as an adaptor for the dynactin complex and phosphoinositide 3-phosphate vesicles, and serves as a scaffold for multiple ion channels including Na^+^/K^+^ ATPase (NKA), Na^+^/Ca^2+^ exchanger type 1 (NCX1), inositol-trisphosphate receptor type 1 (IP3R), and T-type voltage gated calcium channels (VGCC) to the spectrin cytoskeleton network [7–10]. In humans, AnkB variants can lead to AnkB syndrome, which is characterized by a spectrum of cardiac dysfunction [11, 12]. AnkB has also been associated with neurological disorders such as autism spectrum disorder and epilepsy [13–15]. A recent study by our group identified a genetic variant resulting in an amino acid substitution in the membrane binding domain, AnkB p.S646F, which is associated with seizures in addition to cardiac symptoms [16]. The putative molecular and cellular mechanisms underlying neurological symptoms associated with AnkB variants are unknown.

AnkB interacts with Cav2.1, the pore-forming subunit of P/Q type VGCCs, in brain tissues isolated from adult mice [17]. Cav2.1 is detected pre- and post- synaptically, with multiple isoforms (ranging from 160 to 230 kDa, including a 190 kDa dominant isoform) expressed in brain [18, 19]. Cav2.1 plays a role in calcium-triggered neurotransmitter release at the presynaptic membrane, where it interacts with vesicle fusion machinery proteins, such as syntaxin 1A [20–22]. Cav2.1 is also found at the postsynaptic density where it interacts with AMPA receptors [23]. Additionally, Cav2.1 is present on intracellular membranes and is crucial for the fusion of lysosomes to endosomes and autophagosomes [24]. Cav2.1 loss of function mutations lead to several disorders, including episodic ataxia as well as cognitive impairment and epilepsy [25, 26]. In contrast, Cav2.1 gain-of-function is associated with forms of familial migraine [27]. In summary, Cav2.1 plays an important role in maintaining proper neurological function.

Considering the interaction between Cav2.1 and AnkB and the overlap in neurological disorders caused by variants in their respective genes, we explored the relationship between Cav2.1 and AnkB at the subcellular level using transient expression in HEK293T cells and AnkB cortex-specific (Emx1-Cre) heterozygous knockout mice. We found that AnkB and AnkB p.S646F increased overall Cav2.1 expression levels, but did not affect surface Cav2.1 levels in HEK293T cells. Conversely, AnkB p.Q879R increased surface Cav2.1 levels in the presence of accessory subunits α_2_δ_1_ and β_4_, while AnkB p.E1458G decreased surface Cav2.1 in the absence and presence of accessory subunits. In cortical tissues from juvenile mice (postnatal day 30), AnkB KO led to decreased overall Cav2.1 expression levels but no change in synaptosome fraction expression levels. Taken together, our work suggests that AnkB and AnkB variants differentially maintain intracellular and surface pools of Cav2.1.

## Methods

### Plasmids

Wildtype *ANK2* (NCBI accession number NM_020977.3), subcloned into pAcGFP backbone, encodes for the 220 kDa AnkB isoform. *ANK2* c.1937C > T (p.S646F), c.2636A > G (p.Q879R), and c.4373A > G (p.E1458G) point mutations were created using QuikChange II site-directed mutagenesis (Agilent). Constructs were confirmed by DNA sequencing of the entire coding region (Eurofins Genomics). *CACNA1A* (Cav2.1) (NM_012918.3), *CACNA2D1* (α_2_δ_1_) (NM_012919.3)*,* and *CACNB4* (β_4_) (NM_001105733.1) in pcDNA3.1 plasmid were a kind gift from Dr. Terry Snutch (University of British Columbia) [28].

### Cell culture and transfection

Human Embryonic Kidney 293 T (HEK293T) cells from American Type Culture Collection were cultured in Dulbecco’s modified Eagle’s medium supplemented with 10% fetal bovine serum, 100 U/mL penicillin, and 100 μg/mL streptomycin (all from Gibco/Thermo Fisher Scientific). Cells were transfected with 7.5 mM linear polyethylenamine (Polysciences) at a ratio of 1 μg of DNA to 10 μL of PEI, and collected 48 h post-transfection.

### Protein lysate and analysis

HEK293T were washed twice in PBS before addition of cell lysis buffer (25 mM Tris-HCl, 150 mM NaCl, 1 mM EDTA, 1% IGEPAL CA-630, 5% glycerol) supplemented with 10 μL/mL of protease inhibitor cocktail (Millipore Sigma), 0.2 mM PMSF, and 10 μM sodium orthovanadate. Whole cortex, whole hippocampus, and whole cerebellum from C57BL/6 J mice were homogenized in brain lysis buffer (9.1 mM Na_2_HPO_4_, 1.7 mM NaH_2_PO_4_, 150 mM NaCl, 1% IGEPAL CA-630, 0.5% sodium deoxycholate, 0.1% sodium dodecyl sulfate) with the same supplements. Lysate was incubated on ice for 30 min and then centrifuged at 12,000 rpm for 20 min. Supernatants were collected and used for analysis. HEK293T lysates were mixed with sample buffer and reducing agents and stored at -80 °C before running on SDS-PAGE gel. Brain samples with sample buffer and reducing agents were heated to 70 °C for 10 min. Either homemade SDS-PAGE or TGX Stain-Free™ (Bio-Rad) gels was used and transferred overnight onto 0.2 μm pore-size PVDF membrane (Bio-Rad) for Western blotting. Membranes were blocked with 5% skim milk in PBS with 0.1% Tween-20, and probed with primary antibodies. Blots were quantified using ImageJ (http://imagej.nih.gov/ij/).

### GFP immunoprecipitation

GFP Dynabeads™ were prepared by adding 2.5 μg of anti-GFP mouse monoclonal antibody (Roche/Millipore Sigma) to 25 μL of Dynabeads™ Protein G (Invitrogen/Thermo Fisher Scientific) in PBS-T (2.7 mM KCl, 10 mM NaH_2_PO_4_, 1.8 mM KH_2_PO_4_, 137 mM NaCl, 0.02% Tween 20) and incubated at room temperature on a rotator for 30 min. Beads were washed 2x in conjugation buffer (20 mM sodium phosphate (pH 7.4), 150 mM NaCl), cross-linked by resuspending in 5 mM of BS^3^ (Thermo Fisher Scientific) in conjugation buffer, and incubated at room temperature on a rotator for 30 min. Reaction was quenched by adding Tris-HCl (pH 7.5) to a final concentration of 50 mM and incubated at room temperature on a rotator for 15 min. Beads were washed 3x in PBS-T. HEK293T lysates were added to the washed beads and incubated at 4 °C for 2 h on a rotator. Beads were then washed 3x in cell lysis buffer, eluted in 1x sample buffer, and stored at -80 °C before running on SDS-PAGE gel.

### Endogenous immunoprecipitation

As with GFP immunoprecipitation, Dynabeads™ were prepared by adding anti-AnkB antibody (Thermo Fisher Scientific) or mIgG (Jackson ImmunoResearch) to Dynabeads™ at a ratio of 1 μg of antibody to 10 μL of dynabeads™. Mouse cortex was homogenized in IP buffer (9.1 mM Na_2_HPO_4_, 1.7 mM NaH_2_PO_4_, 150 mM NaCl, 0.32 M sucrose, 2 mM EDTA, 0.1% Triton X-100, 0.1% sodium deoxycholate, and 0.1% sodium dodecyl sulfate). Cortex lysates were precleared by incubating with 75 μL of mIgG-Dynabeads™ for 1 h at 4 °C on a rotator. Lysates were removed from the mIgG-Dynabeads™ and added to either 40 μL of AnkB- or mIgG- Dynabeads™ and incubated for 2 h at 4 °C on a rotator. Beads were then washed 3x with IP buffer, eluted with 1x sample buffer with reducing agents and heated at 70 °C for 10 min before running on SDS-PAGE gel.

### Cell surface biotinylation

Transfected HEK293T cells were washed twice with biotinylation buffer (137 mM NaCl, 2.7 mM KCl, 1.8 mM KH_2_PO_4_, 10 mM Na_2_HPO_4_, 0.5 mM MgCl_2_, 1 mM CaCl_2_) and incubated in biontinylation buffer with and without 0.25 mg/mL EZ-Link™ Sulfo-NHS-SS-Biotin (Thermo Fisher Scientific) for 30 min at 4 °C on a rocker. Biotinylation reaction was quenched by adding glycine to the plate to a final concentration of 100 mM (quenching buffer). Cells were then washed twice and then incubated with quenching buffer for 15 min at 4 °C. Cells were then lysed in TBS lysis buffer (50 mM Tris, 150 mM NaCl, 1% IGEPAL CA-630), incubated on ice for 30 min, and spun at 12,000 rpm for 20 min. Lysate was preclear in 50 μL of iminobiotin agarose beads (Pierce/Thermo Fisher Scientific) for 1 h at 4 °C on rotator. Precleared lysate was then added to 50 μL of NeutrAvidin™ agarose beads (Pierce/Thermo Fisher Scientific) for 2 h at 4 °C on rotator. Beads were then washed 4x with TBS lysis buffer, 4x with high salt TBS lysis buffer (50 mM Tris, 300 mM NaCl, 1% IGEPAL CA-630), and finally 2x in 50 mM Tris. Beads were eluted by incubating in 1x sample buffer with reducing agents and stored at -80 °C before running on SDS-PAGE.

### Electrophysiology

Whole cell patch clamp recordings were performed 72 h after transfection using an Axopatch 200B amplifier linked to a computer with pCLAMP 9.2 software. Currents were recorded using the external solution (5 mM BaCl_2_, 137.5 mM CsCl, 1 mM MgCl_2_, 10 mM HEPES and 10 mM Glucose, pH 7.4) and internal pipette solution (130 mM CsCl, 2.5 mM MgCl_2_, 10 mM HEPES, 5 mM EGTA, 3 mM ATP, 0.5 mM GTP, pH 7.4). The current/voltage (I/V) relationship was obtained by applying 250 ms pulses from a holding potential of − 100 mV. Test pulses ranged from − 50 mV to + 50 mV in 5 mV increments. Current density was determined by dividing the peak current by whole cell capacitance and the I/V currents were fitted with a modified Boltzmann equation: I = G_max_ × (V_m_ − V_r_)/(1 + exp.(−(V_m_ − V_1/2_)/k)), where I is the peak current, V_m_ is the membrane potential, V_1/2_ is the voltage for half activation, V_r_ is the reversal potential, and k is the slope factor. Data were analyzed using the Clampfit 10.3 software (Molecular Devices).

### Experimental animals

The animal protocol was approved and the experiments were performed in accordance to the ethical standards set by the University of Victoria’s Animal Care Committee. AnkB^flox/flox^ mice were a kind gift from Dr. Peter Mohler (Ohio State University) and were back-crossed in-house for 5 generations onto a C57BL/6 J background (000664, The Jackson Laboratory) [29]. AnkB^flox/wt^ were crossed with Emx1^IRES-cre^ (#005628, The Jackson Laboratory) to generate control (CTL; AnkB^wt/wt^: Emx1^IRES-cre^) and conditional heterozygous knockout (AnkB^glut+/−^; AnkB^flox/wt^: Emx1^IRES-cre^) littermates. This Emx1^IRES-cre^ drives recombination only in cells that give rise to excitatory neurons and glia of the cerebral cortex and recombination has been shown to occur as early as embryonic day 10.5 [30]. Emx1-Cre turns on in precursors of glutamatergic neurons and glial cells (where AnkB is also expressed; refer to the transcriptome database for cerebral cortex cell by Zhang et al. [31]). Of note, Cav2.1 and syntaxin 1A express at markedly higher levels in neurons than in glial cells, although we still cannot rule out the possibility of indirect effects on neuronal Cav2.1 and syntaxin 1A through partial AnkB KO in astrocytes. Male and female mice were used in the present study; weaning was carried at postnatal day 21. All animals were housed under a 12-h light/dark cycle with water and food ad libitum.

### Synaptosome preparations

Synaptosomes were prepared as previously described [32]. Briefly, P14 and P30 cortices were homogenized in Syn-PER™ (Thermo Fisher Scientific) and a small fraction was saved for analysis. The homogenate was spun at 1200 g for 10 min. The supernatant was transferred to a new tube and spun at 15,000 g for 20 min. The synaptosome pellet was washed by resuspending with Syn- PER™ with 5% DMSO. Sample was stored at -80 °C until needed. Frozen synaptosomes were thawed and spun again at 15,000 g for 20 min. The pellet was then resuspended in Syn-PER™, mixed with sample buffer and reducing agents, heated to 70 °C for 10 min, and analyzed by SDS-PAGE.

### Antibodies

Primary antibodies used included anti-Cav2.1 (1:500, Alomone Labs), anti-GFP rabbit polyclonal (1:2000, Invitrogen/Thermo Fisher Scientific), anti-GAPDH (1:3000, Novus Biologicals), anti-transferrin receptor (1:1000, Invitrogen/Thermo Scientific), anti-CACNA2D1 (1:1000, Abcam), anti-Cavβ_4_ (1:1000, Neuromab), anti-AnkB (1:500, Thermo Fisher Scientific), and anti-syntaxin 1A (1:5000, Millipore Sigma). Secondary antibodies used included horseradish peroxidase (HRP)-conjugated AffiniPure donkey anti-rabbit immunoglobulin and donkey anti-mouse IgG (all at 1:4000; Jackson ImmunoResearch).

### Statistical analysis

Bar graphs display means with standard error of the mean. Analysis used either one-way or two way ANOVA as appropriate. Corrections for multiple comparisons are indicated in figure legends. Data was analyzed using GraphPad Prism version 6.01, with significance denoted as *p* < 0.05 (*), *p* < 0.01 (**), *p* < 0.001 (***), and *p* < 0.0001 (****).

## Results

### Wildtype AnkB and AnkB variants increase overall Cav2.1 expression levels in HEK293T cells

**Fig. 1:**
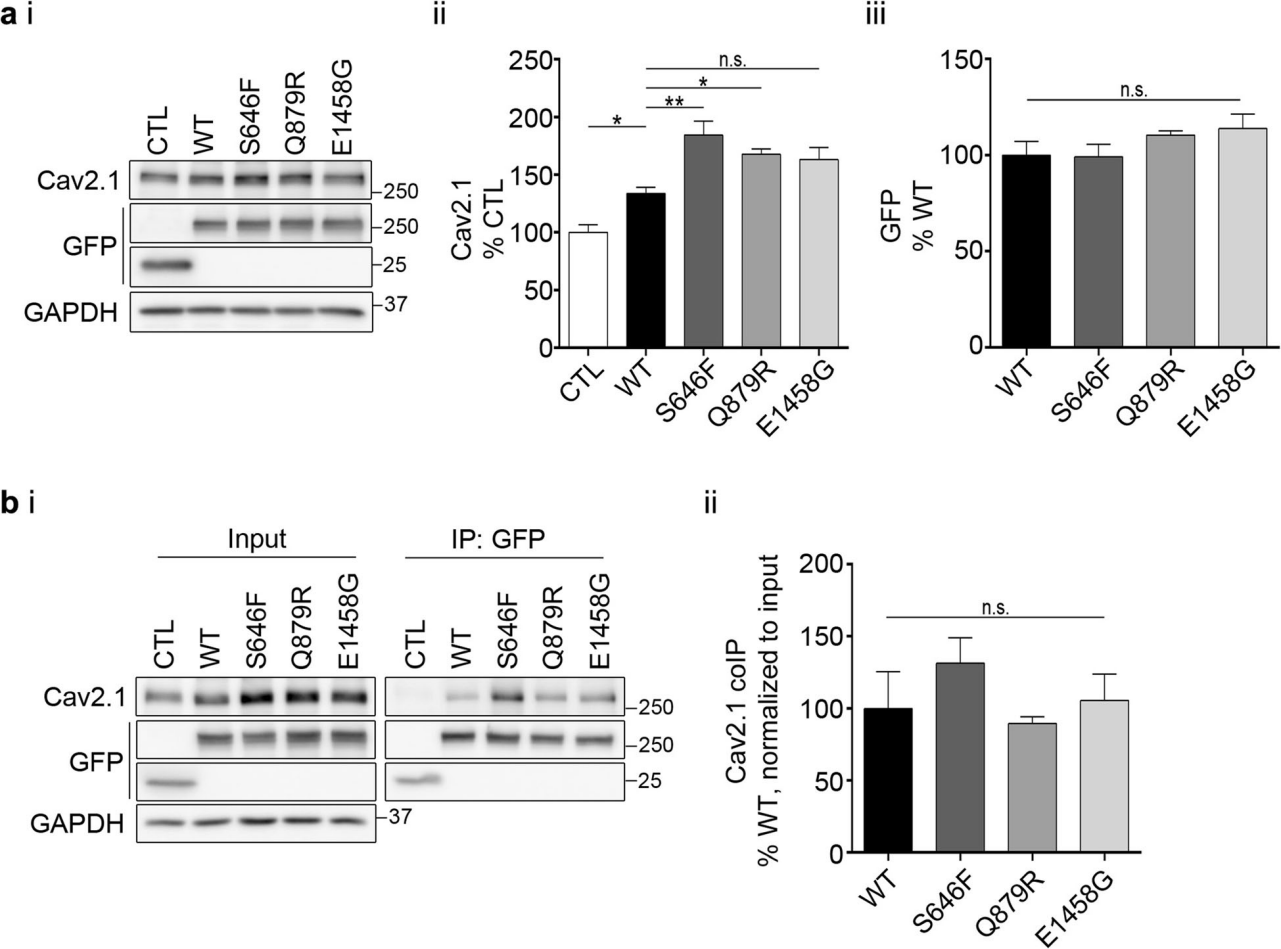
AnkB mutations increased expression of Cav2.1 without altering its binding affinity. (ai) Representative Western blots of whole cell lysates of HEK293T cells transfected with Cav2.1 and GFP control or wildtype or mutant AnkB-GFP. (aii) Quantification of Cav2.1 normalized to GAPDH and expressed as a percentage of control (CTL). One-way ANOVA followed by Sidak’s multiple comparison, *N* = 5, *F*_(4, 20)_ = 15.59, *p* < 0.0001. (aiii) Quantification of GFP normalized to GAPDH and expressed as a percentage of wildtype. One-way ANOVA, *N* = 5, *F*_(3, 16)_ = 1.408, *p* = 0.2770. (bi) Representative co-IP Western blots of transfected HEK293T cells. (bii) Quantification of Cav2.1 co-IP normalized to GFP IP and Cav2.1 input and expressed as a percentage of wildtype. One-way ANOVA, *N* = 3, *F*_(3, 8)_ = 0.9381, *p* = 0.4662. This data is included in the MSc thesis of CSWC, University of Victoria, 2019 found at https://dspace.library.uvic.ca//handle/1828/11053

We first investigated the impact of AnkB expression on overall Cav2.1 expression levels. We performed Western blotting with lysates from HEK293T cells transiently co-expressing Cav2.1 along with wildtype AnkB-GFP or AnkB p.S646F, and two other AnkB variants, p.Q879R and p.E1458G (Fig. 1a). The human variant AnkB p.Q879R was selected for comparison, because the variant resides within the linker region required for proper AnkB localization [33, 34]. In addition, AnkB p.E1458G (referred to as E1425G in previous literature) was selected for its well-characterized cardiac abnormalities as well as loss of binding to NKA, NCX1, and IP3R [9, 35]. Overall Cav2.1 levels increased significantly in cells co-expressing wildtype AnkB-GFP (Fig. 1aii). This effect of AnkB on Cav2.1 levels was even greater in AnkB p.S646F-expressing cells, and cells expressing the other two variants revealed similar effects on Cav2.1 (albeit with differences in statistical significance), suggesting Cav2.1 levels are up-regulated by expression of AnkB as well as its variants. It is important to note that wildtype AnkB-GFP and AnkB-GFP variants were expressed at similar levels (Fig. 1aiii).

### AnkB variants do not alter AnkB-Cav2.1 binding affinity

To investigate whether the increase in overall Cav2.1 expression levels observed with AnkB variants was due to changes in variant binding affinity, we immunoprecipitated AnkB-GFP from HEK293T cells co-expressing Cav2.1 and wildtype AnkB-GFP or an AnkB-GFP variant (Fig. 1b). The quantity of Cav2.1 co-immunoprecipitating with AnkB-GFP or its variants corresponded closely with the overall expression levels in whole cell lysates. As such, no significant differences in co-precipitation of Cav2.1 was observed following normalization to Cav2.1 input levels (Fig. 1bii), suggesting neither AnkB p.S646F nor any of the other variants altered the AnkB-Cav2.1 binding affinity.

### Wildtype AnkB and AnkB variants differentially impact cell surface Cav2.1 levels

**Fig. 2:**
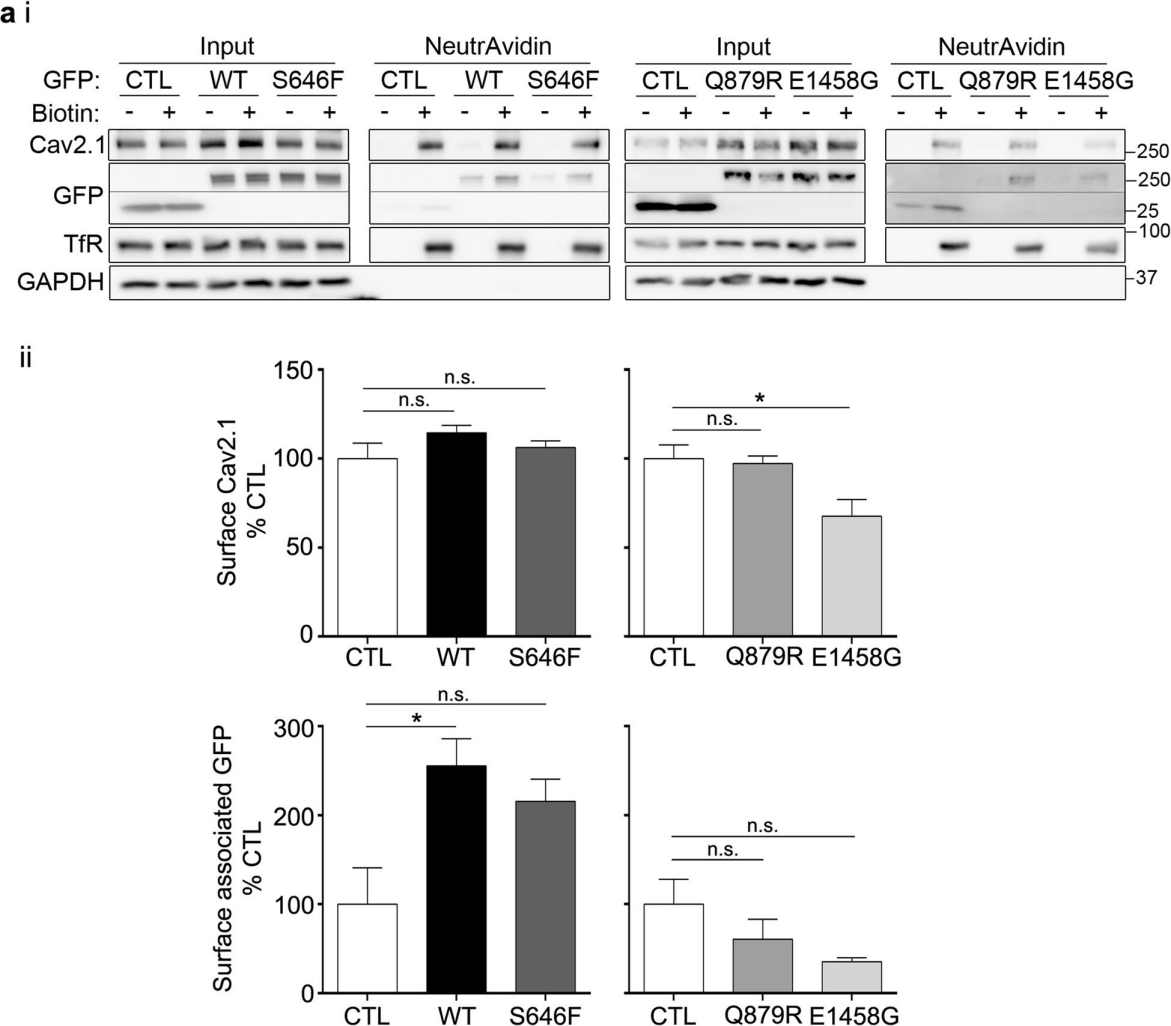
Wildtype AnkB, AnkB p.S646F and AnkB p.Q879R do not impact Cav2.1 surface expression levels. HEK293T cells were co-transfected with Cav2.1, and GFP control, wildtype, or mutant AnkB-GFP. (ai) Representative surface biotinylation Western blots of inputs and NeutrAvidin surface fraction. GAPDH was used as a negative control to ensure intracellular proteins were not biotinylated. Transferrin receptor (TfR) served as a positive pulldown control, (aii) Quantification of surface proteins normalized to surface TfR and expressed as a percentage of GFP control (CTL). One-way ANOVA followed by Dunnett’s multiple comparison, *N* = 3–4, *F*_(2, 8)_ = 1.222, *p* = 0.3443 (Cav2.1: WT,S646F); *N* = 3, *F*_(2, 6)_ = 5.891, *p* = 0.0384 (Cav2.1: Q879R, E1458G); *N* = 3–4, *F*_(2, 8)_ = 5.764, *p* = 0.0282 (GFP: WT, S646F); *N* = 3, *F*_(2, 6)_ = 2.393, *p* = 0.1721 (GFP: Q879R, E1458G). This data is included in the MSc thesis of CSWC, University of Victoria, 2019 found at https://dspace.library.uvic.ca//handle/1828/11053

To determine if the increase in Cav2.1 expression by AnkB led to an increase in cell surface expression, we performed cell surface biotinylation in HEK293T cells transiently expressing Cav2.1 and GFP control, wildtype, or mutant AnkB-GFP. Although AnkB-GFP increased overall Cav2.1 expression levels (Fig. 1a), surprisingly, it did not impact surface Cav2.1 levels (Fig. 2a). Similarly, AnkB p.S646F and AnkB p.Q879R had no significant effect on surface Cav2.1 levels. Conversely, AnkB p.E1458G led to a slight decrease in surface Cav2.1 levels. In addition, we were able to capture cytoplasmic proteins co-precipitating with surface proteins, including a pool of surface-associated AnkB-GFP, by using a gentle lysis buffer in our cell surface biotinylation protocol. We detected a surface associated pool of wildtype AnkB-GFP; however, none of the AnkB variants exhibited a significant surface-associated pool.

### AnkB variants modulate crosstalk between AnkB, Cav2.1, and accessory subunits

**Fig. 3:**
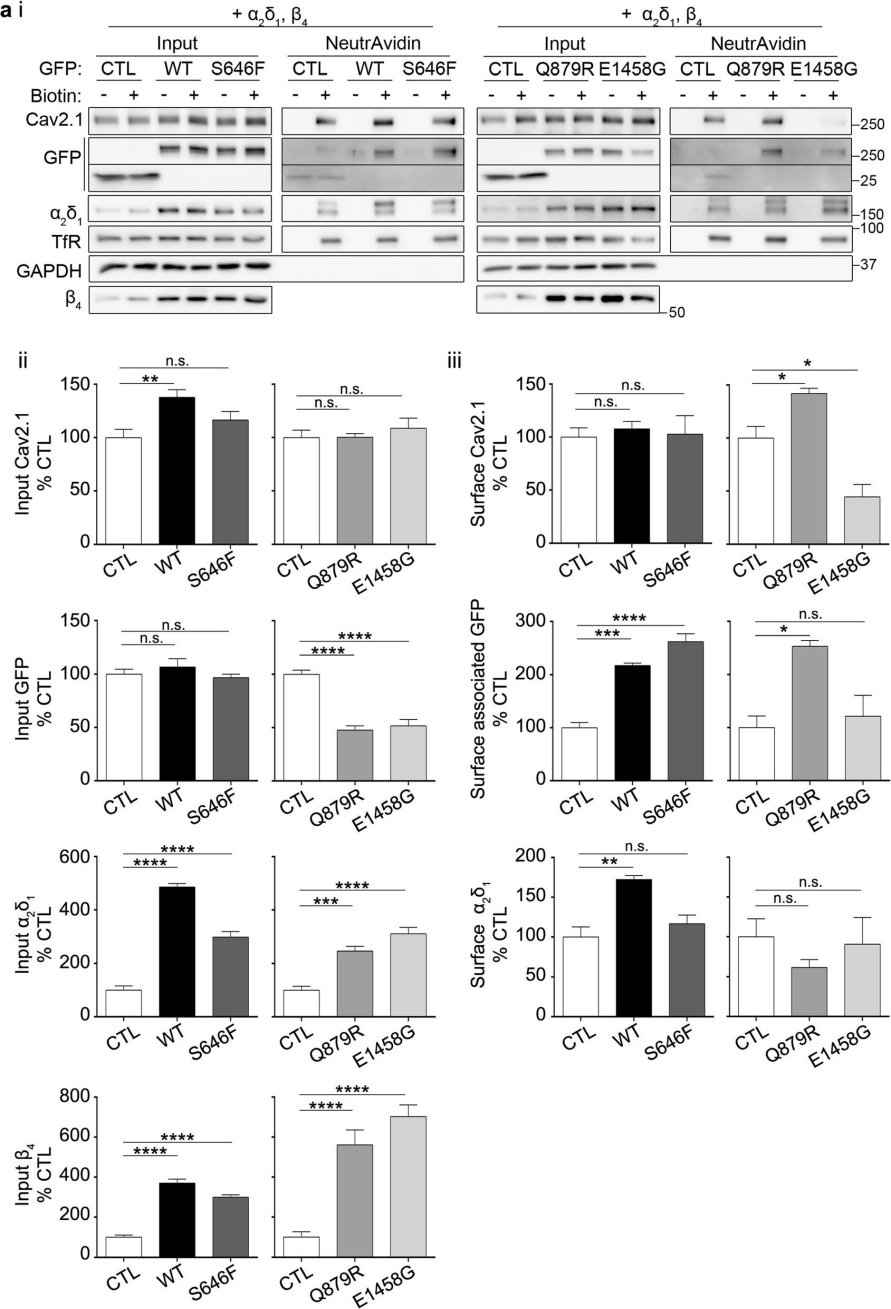
AnkB and its variants differentially impact Cav2.1 expression levels in the presence of auxiliary subunits. HEK293T cells co-transfected with Cav2.1, α_2_δ_1_*,* β_4_, and GFP control, wildtype, or mutant AnkB-GFP (ai) Representative surface biotinylation Western blots of inputs and surface fraction. (aii) Quantification of overall input proteins normalized to GAPDH and expressed as a percentage of GFP control (CTL). One-way ANOVA followed by Dunnett’s multiple comparison, *N* = 6, *F*_(2, 15)_ = 6.072, *p* = 0.0117 (Cav2.1: WT,S646F); *N* = 6, *F*_(2, 15)_ = 0.5018, *p* = 0.6152 (Cav2.1: Q879R, E1458G); *N* = 6, *F*_(2, 15)_ = 0.8088, *p* = 0.4639 (GFP: WT,S646F); *N* = 6, *F*_(2, 15)_ = 36.21, *p* < 0.0001 (GFP: Q879R, E1458G); *N* = 6, *F*_(2, 15)_ = 125.6, *p* < 0.0001 (α_2_δ_1_: WT,S646F); *N* = 6, *F*_(2, 15)_ = 32.05, *p* < 0.0001 (α_2_δ_1_: Q879R, E1458G); *N* = 6, *F*_(2, 15)_ = 90.86, *p* < 0.0001 (β_4_: WT,S646F); *N* = 6, *F*_(2, 15)_ = 30.48, *p* < 0.0001 (β_4_: Q879R, E1458G). (aiii) Quantification of surface proteins normalized to surface TfR and expressed as a percentage of GFP control (CTL). One-way ANOVA followed by Dunnett’s multiple comparison, *N* = 3, *F*_(2, 6)_ = 0.1117, *p* = 0.8961 (Cav2.1: WT,S646F); *N* = 3, *F*_(2, 6)_ = 25.14, *p* = 0.0012 (Cav2.1: Q879R, E1458G); *N* = 3, *F*_(2, 6)_ = 59.18, *p* = 0.0001 (GFP: WT,S646F); *N* = 3, *F*_(2, 6)_ = 9.587, *p* = 0.0135 (GFP: Q879R, E1458G); *N* = 3, *F*_(2, 6)_ = 14.18, *p* = 0.0053 (α_2_δ_1_: WT,S646F); *N* = 3, *F*_(2, 6)_ = 0.6860, *p* = 0.5392 (α_2_δ_1_: Q879R, E1458G). A portion of this data is included in the MSc thesis of CSWC, University of Victoria, 2019 found at https://dspace.library.uvic.ca//handle/1828/11053

Trafficking and regulation of Cav2.1 is affected by accessory α_2_δ and β subunits, which affect expression levels, ER exit, and surface localization of the pore-forming Cav subunit [36, 37]. To determine if these accessory subunits impact AnkB regulation of overall and surface Cav2.1 expression levels, we performed surface biotinylation on HEK293T expressing AnkB, Cav2.1 as well as α_2_δ_1_ and β_4_ (Fig. 3a). In the presence of α_2_δ_1_ and β_4,_ only wildtype AnkB increased overall Cav2.1 levels (Fig. 3aii). In addition, both overall α_2_δ_1_ and β_4_ levels were dramatically increased in the presence of wildtype AnkB and AnkB variants. Conversely, overall AnkB p.Q879R and p.E1458G levels decreased in the presence of α_2_δ_1_ and β_4_. There was still no effect of wildtype AnkB or AnkB p.S646F on surface Cav2.1 levels in the presence of α_2_δ_1_ and β_4_, and AnkB p.E1458G continued to decrease surface Cav2.1 levels (Fig. 3aiii). Conversely, AnkB p.Q879R increased Cav2.1 surface levels in the presence of α_2_δ_1_ and β_4._ Moreover, α_2_δ_1_ and β_4_ expression promoted surface-association of AnkB p.S646F and p.Q879R variants, which did not occur in the absence of accessory subunits. Furthermore, wildtype AnkB increased α_2_δ_1_ surface levels, whereas AnkB variants did not. Together these results suggest that AnkB variants modulate crosstalk between AnkB, Cav2.1 and accessory subunits.

### AnkB and AnkB variants increase Cav2.1-based VGCC peak current density

**Fig. 4:**
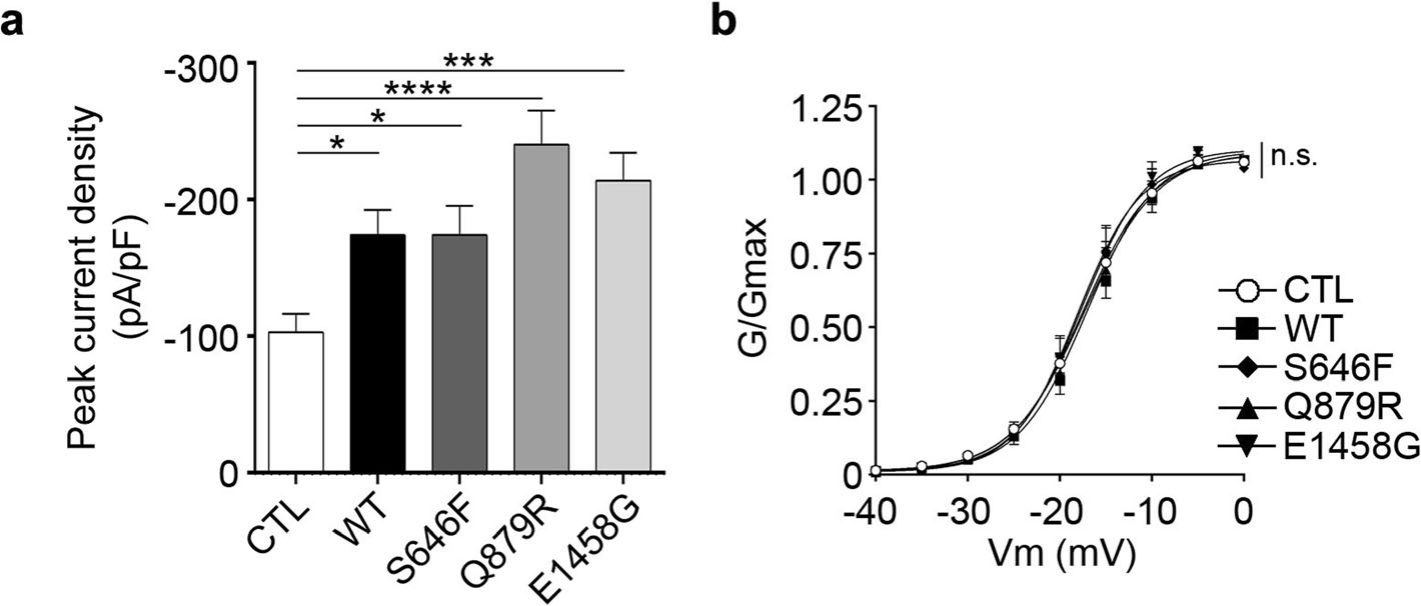
AnkB and its variants increase peak current density. HEK293T cells were co-transfected with Cav2.1, α_2_δ_1_*,* β_4_, and GFP control, wildtype, or mutant AnkB-GFP. (**a**) Peak current density was determined by dividing the peak current by whole cell capacitance. One-way ANOVA followed by Dunnett’s multiple comparison, *F*_(4,151)_ = 6.465, *p* < 0.0001 (**b**) Normalized G/Gmax vs. voltage curve fitted using Boltzmann’s equation. One-way ANOVA *F*_(4, 50)_ = 0.0022, *p* > 0.9999. This data is included in the MSc thesis of CSWC, University of Victoria, 2019 found at https://dspace.library.uvic.ca//handle/1828/11053

We next investigated the impact of wildtype AnkB and AnkB variants on Cav2.1-based VGCC properties. We measured peak current density using whole-cell patch clamp in HEK293T cells expressing wildtype AnkB-GFP or AnkB-GFP variants along with Cav2.1, and accessory subunits (α_2_δ_1_ and β_4_). Expression of wildtype AnkB and AnkB variants all resulted in an increase in peak current density, with the greatest increase by AnkB p.Q879R (Fig. 4a). Neither wildtype nor mutant AnkB significantly affected the voltage dependence of activation of Cav2.1 channels (Fig. 4b).

### AnkB alters a non-synaptic pool of Cav2.1

**Fig. 5:**
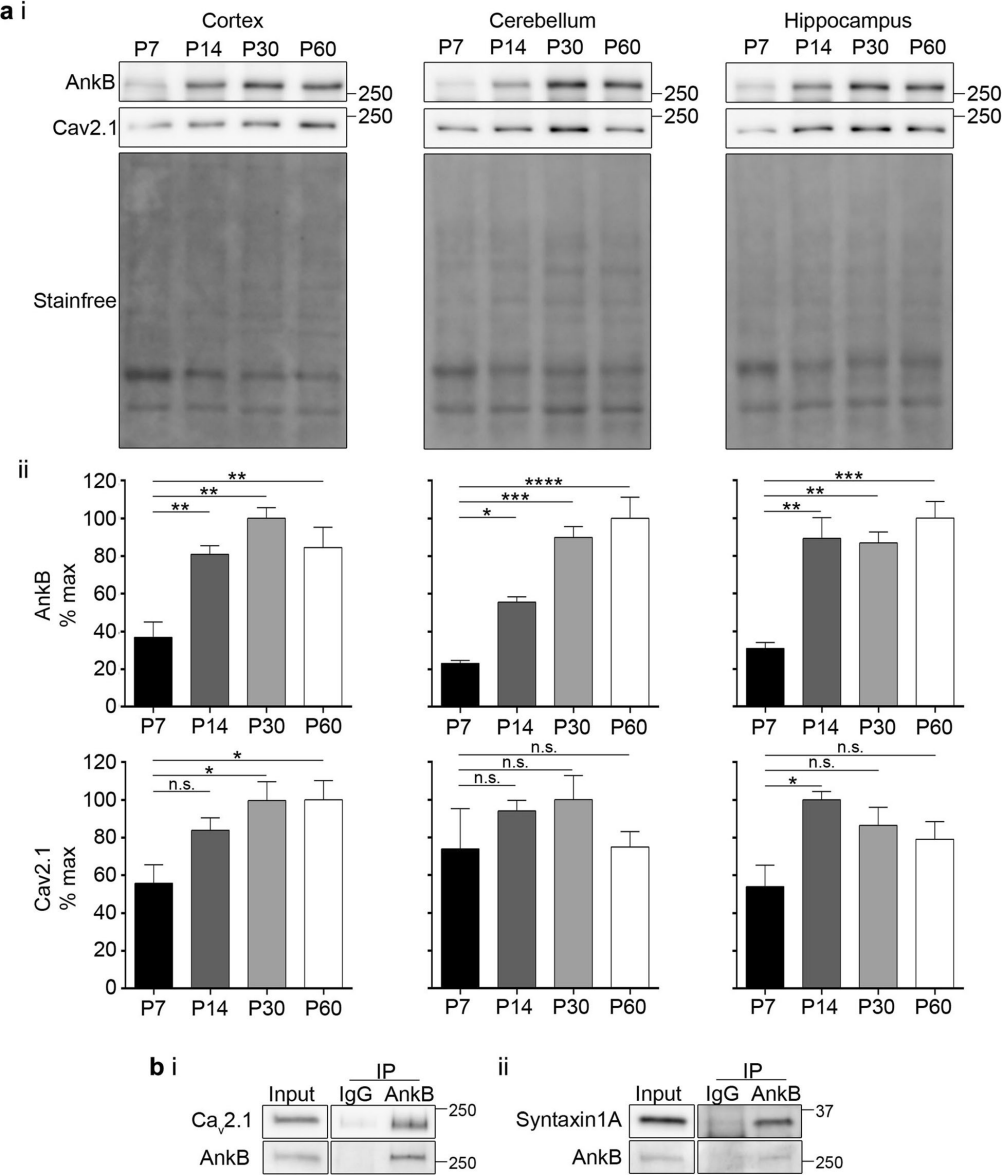
Both AnkB and Cav2.1 show similar increase in expression during early cortical development. (ai) Representative Western blots of AnkB and Cav2.1 in mice cortex, hippocampus, and cerebellum from P7 to P60. (aii) Quantification of AnkB and Cav2.1 normalized to stain-free. One-way ANOVA followed by Dunnett’s multiple comparison, *N* = 3, *F*_(3, 8)_ = 12.52, *p* = 0.0022 (cortex, AnkB); *F*_(3, 8)_ = 4.945, *p* = 0.0314 (cortex, Cav2.1); *F*_(3, 8)_ = 28.89, *p* = 0.0001 (cerebellum, AnkB); *F*_(3, 8)_ = 0.9790, *p* = 0.4494 (cerebellum, Cav2.1); *F*_(3, 8)_ = 15.70, *p* = 0.0010 (hippocampus, AnkB); *F*_(3, 8)_ = 4.507, *p* = 0.0393 (hippocampus, Cav2.1). (bi-ii) Representative Western blots of inputs and control IgG or AnkB immunoprecipitation from P14 mouse cortex. *N* = 3. This data is included in the MSc thesis of CSWC, University of Victoria, 2019 found at https://dspace.library.uvic.ca//handle/1828/11053

To further investigate the role of AnkB in the regulation of Cav2.1 expression in mouse brain, we first determined their postnatal developmental expression profiles from postnatal day 7 (P7) to P60 in wildtype mice (Fig. 5a). In the cortex and hippocampus, both AnkB and Cav2.1 expression levels increased markedly between P7 and P14. Conversely, in the cerebellum, AnkB levels increased gradually over postnatal development, while Cav2.1 levels were high from P7 onwards. Cav2.1 is known to interact with AnkB in adult mouse cortex [17]. To determine whether Cav2.1 and AnkB also interact during early postnatal development, we immunoprecipitated AnkB from P14 wildtype mouse cortex (Fig. 5b). Our results confirmed that AnkB interacts with Cav2.1 in P14 mouse cortex. We also examined syntaxin 1A, an important SNARE protein that interacts with the *syn*aptic *pr*otein *int*eraction (synprint) site within the Cav2.1 II-III linker which is also the locus of the AnkB interaction [17, 20], and discovered that syntaxin 1A was also immunoprecipitated by AnkB.

**Fig. 6:**
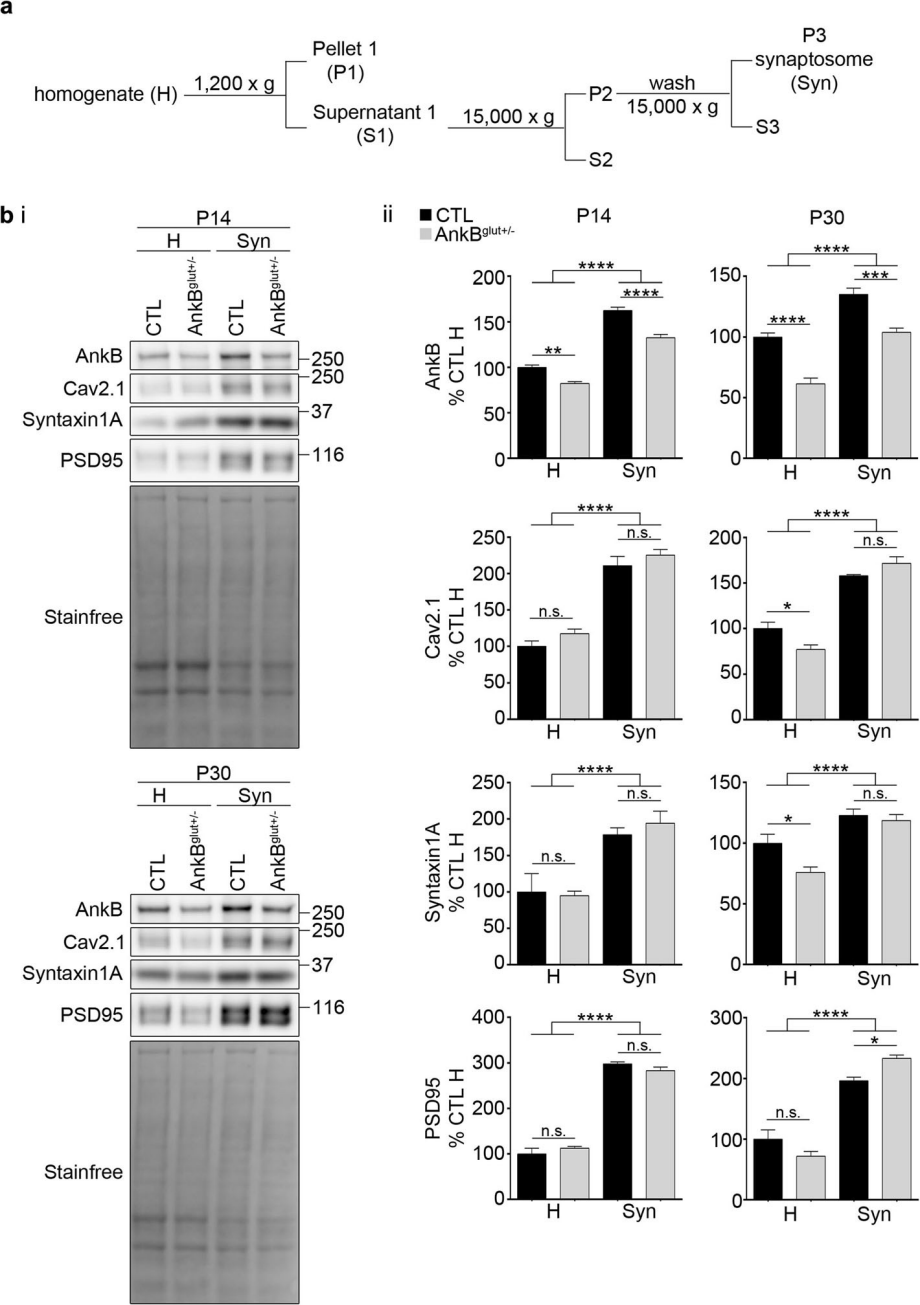
Decreased Cav2.1 expression levels in whole cortex homogenate of P30 AnkB^glut+/−^. (a) Flow chart of synaptosome preparation procedure. (bi) Representative Western blots of P14 and P30 homogenate (H) and synaptosome (Syn) fraction from control (CTL; AnkB^wt/wt^: Emx1^IRES-cre^) and AnkB conditional heterozygous knockout (AnkB^glut+/−^; AnkB^flox/wt^: Emx1^IRES-cre^) cortices. (bii) Quantification of immunoblots normalized to stain-free and expressed as a percentage of control homogenate. Two-way ANOVA followed by Sidak’s multiple comparison, *N* = 5, interaction *F*_(1, 16)_ = 4.047, *p* = 0.0614, genotype: *F*_(1, 16)_ = 59.15, *p* < 0.0001, fraction *F*_(1, 16)_ = 332.4, *p* < 0.0001 (P14 AnkB); *N* = 5, interaction *F*_(1, 16)_ = 0.7049, *p* = 0.4135, genotype: *F*_(1, 16)_ = 68.89, *p* < 0.0001, fraction *F*_(1, 16)_ = 85.56, *p* < 0.0001 (P30 AnkB); *N* = 5, interaction *F*_(1, 16)_ = 0.0269, *p* = 0.8716, genotype: *F*_(1, 16)_ = 3.025, *p* = 0.1012, fraction *F*_(1, 16)_ = 143.3, *p* < 0.0001 (P14 Cav2.1); *N* = 5, interaction *F*_(1, 16)_ = 10.47, *p* = 0.0052, genotype: *F*_(1, 16)_ = 0.6942, *p* = 0.4170, fraction *F*_(1, 16)_ = 181.7, *p* < 0.0001 (P30 Cav2.1); *N* = 5, interaction *F*_(1, 16)_ = 0.4101, *p* = 0.5310, genotype: *F*_(1, 16)_ = 0.1144, *p* = 0.7396, fraction *F*_(1, 16)_ = 29.99, *p* < 0.0001 (P14 syntaxin 1A); *N* = 5, interaction *F*_(1, 16)_ = 3.163, *p* = 0.0943, genotype: *F*_(1, 16)_ = 6.345, *p* = 0.0228, fraction *F*_(1, 16)_ = 34.34, *p* < 0.0001 (P30 syntaxin 1A); *N* = 4, interaction *F*_(1, 12)_ = 2.869, *p* = 0.1161, genotype: *F*_(1, 12)_ = 0.013, *p* = 0.9108, fraction *F*_(1, 12)_ = 514.9, *p* < 0.0001 (P14 PSD95); *N* = 4, interaction *F*_(1, 12)_ = 11.33, *p* = 0.0056, genotype: *F*_(1, 12)_ = 0.2076, *p* = 0.6568, fraction *F*_(1, 12)_ = 117.8, *p* < 0.0001 (P30 PSD95). This data is included in the MSc thesis of CSWC, University of Victoria, 2019 found at https://dspace.library.uvic.ca//handle/1828/11053

Based on the coordinated developmental increase in AnkB and Cav2.1 levels and the early postnatal AnkB-Cav2.1 interaction, we next investigated the impact of AnkB KO on Cav2.1 levels in cortical tissue. To do this we used cortical tissue isolated from control mouse (CTL; AnkB^wt/wt^: Emx1^IRES-cre^) and AnkB conditional heterozygous knockout (AnkB^glut+/−^; AnkB^flox/wt^: Emx1^IRES-cre^) littermates and performed synaptosome fractionation (Fig. 6a) yielding homogenates (H; consisting of whole cortical lysate) and synaptosomes (Syn). Confirming successful partial deletion of *ANK2*, AnkB levels were lower in AnkB^glut+/−^ than control cortical tissue homogenate (Fig. 6b). We validated synaptic compartment enrichment in the synaptosome fractions using an antibody against post-synaptic density protein 95 (PSD95), a key excitatory post-synaptic scaffold [38]. We compared Cav2.1 expression in synaptosome and homogenate fractions from P14 and P30 cortices using Western blot analysis. The levels of AnkB and Cav2.1 were enriched in control and AnkB^glut+/−^ synaptosome fractions at P14 and P30, confirming localization to synaptic compartments (Fig. 6c) [18, 39–41]. AnkB levels were lower in AnkB^glut+/−^ synaptosomes than control synaptosomes, whereas Cav2.1 levels were unchanged, suggesting that the Cav2.1 synaptic pool remained unaffected. However, Cav2.1 cortical homogenate expression levels were lower in AnkB^glut+/−^ than control at P30. We also investigated the consequences of partial AnkB KO on the expression levels of syntaxin 1A. Similarly, syntaxin 1A cortical homogenate expression levels from P30 AnkB^glut+/−^ were lower than control, whereas there was no change in syntaxin 1A synaptosome levels between groups. These decreases in Cav2.1 and syntaxin 1A expression were only seen at P30 but not at P14. Unlike Cav2.1 or syntaxin 1A, AnkB^glut+/−^ led to a higher level of PSD95 in P30 synaptosome than wildtype control.

## Discussion

Our results presented here build on our knowledge of the relationship between the scaffold protein AnkB and the neuronal, endothelial, and pancreatic Cav2.1 protein, with important implications for understanding the mechanisms of extra-cardiac disease resulting from AnkB variants. Together our results suggest that AnkB regulates an intracellular pool of Cav2.1, a pool that may play an important role in neuronal homeostasis. Notably our findings suggest that this role is modulated by expression of AnkB variants associated with disease. Therefore dysregulation of Cav2.1 may present a possible mechanism for the pathogenicity of AnkB variants in the context of nervous system symptoms, such as seizure for AnkB p.S646F.

Taken together, our cellular expression system and cortical tissue experiments point to a new role for AnkB in the regulation of intracellular Cav2.1 levels. We found that overall Cav2.1 expression levels were higher in HEK293T cells transiently expressing AnkB than in control cells. Similarly, partial loss of AnkB in mouse cortex (AnkB^glut+/−^) led to an overall decrease in Cav2.1 expression levels with no change in the synaptosome fraction. Notably, although we observed reduced AnkB expression in AnkB^glut+/−^ in cortical homogenates at P14, we did not observe a decrease in Cav2.1 until P30, suggesting AnkB regulation of Cav2.1 changes over the course of postnatal development. The precise role of AnkB in regulating Cav2.1 levels is beyond the scope of this study; for example, AnkB could regulate the production of Cav2.1 through indirect mechanisms, or AnkB could stabilize Cav2.1, protecting it from degradation. A role for AnkB in regulating expression levels of Cav2.1 is consistent with previous studies demonstrating it regulates the expression levels of other channel/receptor proteins, like NCX1 and IP3R [42, 43]. Notably, the p.E1458G variant led to loss of binding to NCX1, NKA, and IP3R [9], but retains its binding to Cav2.1. NCX1 and IP3R binds to repeats 16–18 and 22–24 respectively, while Cav2.1 interaction has only been narrowed down to the membrane binding domain [17, 42, 43]. Potentially, the p.E1458G variant abolished binding to ankyrin repeats specific to the NCX1 and IP3R interactions without affecting the ankyrin repeats involved in Cav2.1 binding. Overall, these findings support a role for AnkB in regulating non-synaptic Cav2.1 expression levels in neurons.

Regulation of Cav2.1 expression levels is likely critical for the development and maintenance of proper neuronal and pancreatic function. In neurons, Cav2.1 is localized not only to nerve terminals for neurotransmitter release, but also in dendrites and the cell body [18, 19, 44]. Previous work on related VGCCs in different systems points to the possibility that intracellular Cav2.1 may serve as a reserve source, which under certain conditions or stimuli could be transported to the plasma membrane. For example, in bag cell neurons in Aplysia, Cav2 have been detected in intracellular punctae trafficking to distal lammellipodium upon PKC activation [45]. Similarly, a pool of N-type calcium channels, another presynaptic VGCC containing a synprint site [46], can be found on secretory granules of IMR32 neuroblastoma cells that are translocated to the plasma membrane upon stimulation [47]. Intracellular Cav2.1 could additionally or alternatively play a direct role in endomembrane compartment regulation. For example, a recent report revealed lysosomal localization of Cav2.1 in cultured cerebellar neurons, where it is required for lyso-endosome fusion/autophagosomal maturation [24]. The authors of the study proposed that lysosomal Cav2.1 enables calcium efflux triggering SNARE mediated fusion of the lysosome to the endosome. Notably, in the present study, we found that the SNARE protein syntaxin 1A co-precipitates with AnkB, suggesting that it interacts either directly or indirectly through Cav2.1. We also found that partial loss of AnkB had a similar effect on syntaxin 1A expression levels as it did on Cav2.1 levels. Although it is possible that AnkB interacts with syntaxin 1A separately from Cav2.1, considering the closely linked functions of Cav2.1 and syntaxin 1A, it is reasonable to speculate that AnkB, syntaxin 1A, and Cav2.1 form a complex. As AnkB is a well-known scaffolding protein, it may also serve here to stabilize Cav2.1 to the SNARE machinery. The interactions between AnkB, Cav2.1, and syntaxin 1A could serve to regulate intracellular Cav2.1 localization and function, and the regulation of the intracellular pool of Cav2.1 may be an important component of neuronal homeostasis.

**Fig. 7:**
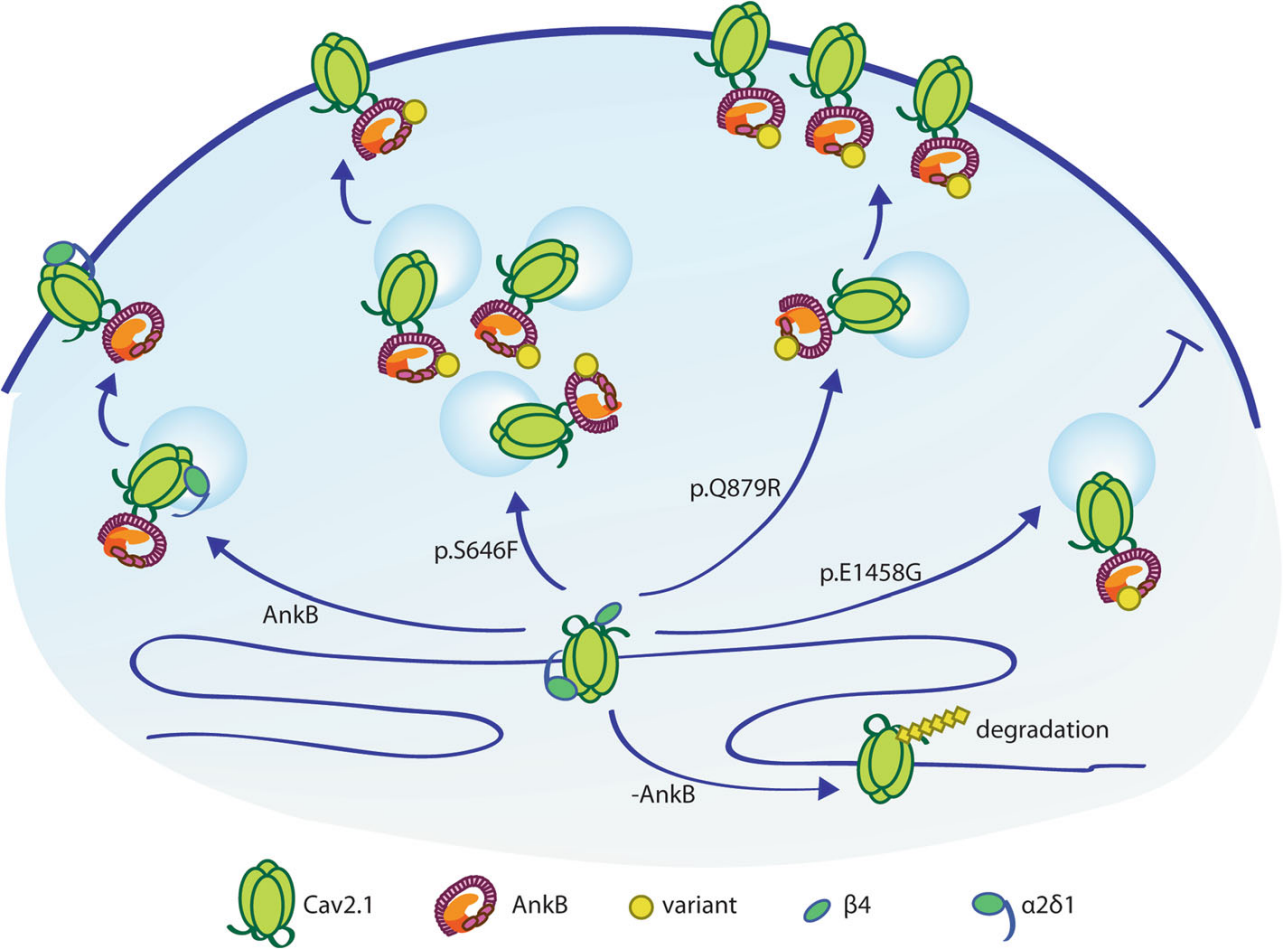
Working model of AnkB’s regulation of Cav2.1. In the absence of AnkB, Cav2.1 is expressed at a low level in the cell. In the presence of wildtype AnkB, Cav2.1 may be protected from degradation and therefore has higher intracellular levels while maintaining low surface levels. With the AnkB p.S646F variant, Cav2.1 is expressed at an even higher level intracellularly, but maintains the same surface expression levels. The AnkB p.Q879R variant increases surface Cav2.1, while AnkB p.E1458G decreases surface Cav2.1 levels. AnkB wildtype, p.S646F, and p.Q879R all had a surface-associated pool in the presence of Cav accessory subunits. There was no significant surface-associated pool with AnkB p.E1458G. This figure is included in the MSc thesis of CSWC, University of Victoria, 2019 found at https://dspace.library.uvic.ca//handle/1828/11053

In parallel to studying the effects of wildtype AnkB, as discussed above, we also compared the effects of AnkB p.S646F, p.Q879R, and p.E1458G, on overall and surface Cav2.1 expression levels (Fig. 7). Notably we found that each AnkB variant differentially affected surface Cav2.1 expression levels in the presence of accessory subunits: AnkB p.E1458G caused a decrease, AnkB p.Q879R caused an increase, while wildtype AnkB and AnkB p.S646F had no effect. Unexpectedly, AnkB and AnkB p.S646F, p.Q879R, and p.E1458G all increased Cav2.1 peak current density. While these findings are difficult to reconcile, they suggest that AnkB and its variants impact on channel activity via mechanisms that are independent of the effects on trafficking. With the exception of AnkB p.Q879R, neither wildtype nor the other AnkB variants were able to increase Cav2.1 surface expression, even in the presence of accessory subunits. Under our experimental conditions, it is possible that we are observing a “ceiling” effect such as that observed for Cav2.2 in rat superior cervical ganglion neurons, in which there was a maximum surface expression observed [48]. Intriguingly, expression of AnkB and AnkB variants led to substantial increases in overall α_2_δ_1_ and β_4_ expression levels, but again, paradoxically, were not able to increase Cav2.1 surface expression, with the exception of AnkB p.Q879R. It is reasonable to speculate that the increased current density associated with AnkB or AnkB variant expression are due to as yet undetermined effects on channel properties, although the precise mechanisms are beyond the scope of the current study. Additionally, AnkB variants differentially affected surface association of AnkB itself, but the significance of these findings remains unclear. Overall, our results suggest that complex interplay between AnkB variants, Cav2.1, α_2_δ_1_ and β_4,_ along with other, as yet unidentified, components differentially regulate overall and surface Cav2.1 expression levels and channel function.

Together our findings provide evidence that AnkB regulates an intracellular pool of Cav2.1 and that certain AnkB variants can also alter surface Cav2.1 levels. This discovery sheds new light on the complex regulation of Cav2.1 necessary for proper neuronal function and will be important for understanding nervous system manifestations associated with AnkB variants.

## Data Availability

The datasets used and/or analyzed during the current study are available from the corresponding author on reasonable request.

## Abbreviations

AnkB: Ankyrin B
AnkB^flox/wt^: Emx1^IRES-cre^
AnkB^glut+/−^: AnkB conditional heterozygous knockout
GAPDH: Glyceraldehyde 3-phosphate dehydrogenase
IP3R: Inositol-trisphosphate receptor
NCX1: Na^+^/Ca^2+^ exchanger type 1
NKA: Na^+^/K^+^ ATPase
SNARE: Soluble N-ethylmaleimide-sensitive fusion protein attachment protein receptors
TfR: Transferrin Receptor
VGCC: Voltage gated calcium channels

## References

[1] Scotland P, Zhou D, Benveniste H, Bennett V (1998). Nervous system defects of AnkyrinB(−/−) mice suggest functional overlap between the cell adhesion molecule L1 and 440-kD AnkyrinB in premyelinated axons. J Cell Biol.

[2] Smith KR, Ankyrins PP (2018). Roles in synaptic biology and pathology. Mol Cell Neurosci.

[3] Bennett V., Healy J. (2009). Membrane Domains Based on Ankyrin and Spectrin Associated with Cell-Cell Interactions. Cold Spring Harbor Perspectives in Biology.

[4] Mohler PJ, Healy JA, Xue H, Puca AA, Kline CF, Allingham RR (2007). Ankyrin-B syndrome: enhanced cardiac function balanced by risk of cardiac death and premature senescence. PLoS One.

[5] Koenig SN, Mohler PJ (2017). The evolving role of ankyrin-B in cardiovascular disease. Hear Rhythm.

[6] Lorenzo DN, Healy JA, Hostettler J, Davis J, Yang J, Wang C (2015). Ankyrin-B metabolic syndrome combines age-dependent adiposity with pancreatic β cell insufficiency. J Clin Invest.

[7] Lorenzo DN, Badea A, Davis J, Hostettler J, He J, Zhong G (2014). A PIK3C3-Ankyrin-B-dynactin pathway promotes axonal growth and multiorganelle transport. J Cell Biol.

[8] Lencesova L, O’Neill A, Resneck WG, Bloch RJ, Blaustein MP (2004). Plasma membrane-cytoskeleton-endoplasmic reticulum complexes in neurons and astrocytes. J Biol Chem.

[9] Mohler PJ, Davis JQ, Bennett V (2005). Ankyrin-B coordinates the Na/K ATPase, Na/ca exchanger, and InsP3 receptor in a cardiac T-tubule/SR microdomain. PLoS Biol.

[10] Garcia-Caballero A, Zhang F, Hodgkinson V, Huang J, Chen L, Souza IA (2018). T-type calcium channels functionally interact with spectrin (α/β) and ankyrin B. Mol Brain.

[11] Mohler PJ, Splawski I, Napolitano C, Bottelli G, Sharpe L, Timothy K (2004). A cardiac arrhythmia syndrome caused by loss of ankyrin-B function. Proc Natl Acad Sci U S A.

[12] Mohler PJ, Le Scouarnec S, Denjoy I, Lowe JS, Guicheney P, Caron L (2007). Defining the cellular phenotype of “ankyrin-B syndrome” variants: human ANK2 variants associated with clinical phenotypes display a spectrum of activities in cardiomyocytes. Circulation..

[13] Iossifov I, O’Roak BJ, Sanders SJ, Ronemus M, Krumm N, Levy D (2014). The contribution of de novo coding mutations to autism spectrum disorder. Nature..

[14] De RS, He X, Goldberg AP, Poultney CS, Samocha K, Cicek A (2014). Synaptic, transcriptional, and chromatin genes disrupted in autism. Nature..

[15] Guo W, Shang D-M, Cao J-H, Feng K, He Y-C, Jiang Y (2017). Identifying and analyzing novel epilepsy-related genes using random walk with restart algorithm. Biomed Res Int.

[16] Swayne LA, Murphy NP, Asuri S, Chen L, Xu X, McIntosh S (2017). Novel variant in the ANK2 membrane-binding domain is associated with Ankyrin-B syndrome and structural heart disease in a first nations population with a high rate of long QT syndrome. Circ Cardiovasc Genet.

[17] Kline CF, Scott J, Curran J, Hund TJ, Mohler PJ (2014). Ankyrin-B regulates Cav2.1 and Cav2.2 channel expression and targeting. J Biol Chem.

[18] Fu S-J, Jeng C-J, Ma C-H, Peng Y-J, Lee C-M, Fang Y-C (2017). Ubiquitin ligase RNF138 promotes episodic Ataxia type 2-associated aberrant degradation of human ca _v_ 2.1 (P/Q-type) calcium channels. J Neurosci.

[19] Sakurai T, Westenbroek RE, Rettig J, Hell J, Catterall WA (1996). Biochemical-properties and subcellular-distribution of the bi and rba isoforms of alpha(1a) subunits of brain calcium channels. J Cell Biol.

[20] Rettig J, Sheng ZH, Kim DK, Hodson CD, Snutch TP, W A C (1996). Isoform-specific interaction of the alpha1A subunits of brain Ca2+ channels with the presynaptic proteins syntaxin and SNAP-25. Proc Natl Acad Sci U S A.

[21] Catterall WA (2011). Voltage-gated calcium channels. Cold Spring Harb Perspect Biol.

[22] He R, Zhang J, Yu Y, Jizi L, Wang W, Li M (2018). New insights into interactions of presynaptic Calcium Channel subtypes and SNARE proteins in neurotransmitter release. Front Mol Neurosci.

[23] Kang M-G, Chen C-C, Wakamori M, Hara Y, Mori Y, Campbell KP (2006). A functional AMPA receptor-calcium channel complex in the postsynaptic membrane. Proc Natl Acad Sci U S A.

[24] Tian X, Gala U, Zhang Y, Shang W, Nagarkar Jaiswal S, di Ronza A (2015). A voltage-gated Calcium Channel regulates lysosomal fusion with endosomes and autophagosomes and is required for neuronal homeostasis. PLoS Biol.

[25] Damaj L, Lupien-meilleur A, Lortie A, Riou É, Ospina LH, Gagnon L (2015). CACNA1A haploinsufficiency causes cognitive impairment , autism and epileptic encephalopathy with mild cerebellar symptoms. Eur J Hum Genet.

[26] Heyne HO, Singh T, Stamberger H, Jamra RA, Caglayan H, Craiu D (2018). De novo variants in neurodevelopmental disorders with epilepsy. Nat Genet.

[27] Pietrobon D (2010). Insights into migraine mechanisms and CaV2.1 calcium channel function from mouse models of familial hemiplegic migraine. J Physiol.

[28] Snutch TP, Leonard JP, Gilbert MM, Lester HA, Davidson N (1990). Rat brain expresses a heterogeneous family of calcium channels. Proc Natl Acad Sci.

[29] Smith Sakima A., Sturm Amy C., Curran Jerry, Kline Crystal F., Little Sean C., Bonilla Ingrid M., Long Victor P., Makara Michael, Polina Iuliia, Hughes Langston D., Webb Tyler R., Wei Zhiyi, Wright Patrick, Voigt Niels, Bhakta Deepak, Spoonamore Katherine G., Zhang Chuansheng, Weiss Raul, Binkley Philip F., Janssen Paul M., Kilic Ahmet, Higgins Robert S., Sun Mingzhai, Ma Jianjie, Dobrev Dobromir, Zhang Mingjie, Carnes Cynthia A., Vatta Matteo, Rasband Matthew N., Hund Thomas J., Mohler Peter J. (2015). Dysfunction in the βII Spectrin–Dependent Cytoskeleton Underlies Human Arrhythmia. Circulation.

[30] Gorski JA, Talley T, Qiu M, Puelles L, Rubenstein JLR, Jones KR (2002). Cortical excitatory neurons and glia, but not GABAergic neurons, are produced in the Emx1-expressing lineage. J Neurosci.

[31] Zhang Y, Chen K, Sloan SA, Bennett ML, Scholze AR, O’Keeffe S (2014). An RNA-sequencing transcriptome and splicing database of glia, neurons, and vascular cells of the cerebral cortex. J Neurosci.

[32] Sanchez-arias JC, Liu M, Choi CSW, Ebert SN, Brown CE, Swayne A (2019). Pannexin 1 regulates network ensembles and dendritic spine development in cortical neurons. eNeuro..

[33] He M, Tseng WC, Bennett V (2013). A single divergent exon inhibits ankyrin-B association with the plasma membrane. J Biol Chem.

[34] Exome Variant Server NHLBI GO Exome Sequencing Project (ESP), Seattle, WA (URL: http://evs.gs.washington.edu/EVS/) Accessed 22 May 2019.

[35] Mohler PJ, Schott J-J, Gramolini a O, Dilly KW, Guatimoisim S, DuBell WH (2003). Ankyrin-B mutations causes type 4 long-QT cardiac arrhythmia and sudden cardiac death. Nature..

[36] Weiss N, Zamponi GW (2017). Trafficking of neuronal calcium channels. Neuronal Signal.

[37] Buraei Z, Yang J (2010). The β subunit of voltage-gated ca 2+ channels. Physiol Rev.

[38] El-Husseini A-D, Schnell E, Chetkovich DM, Nicoll RA, Bredt DS (2000). PSD-95 involvement in maturation of excitatory synapses. Science (80- ).

[39] Collins MO, Husi H, Yu L, Brandon JM, Anderson CNG, Blackstock WP (2006). Molecular characterization and comparison of the components and multiprotein complexes in the postsynaptic proteome. J Neurochem.

[40] Jordan Bryen A., Fernholz Brian D., Boussac Muriel, Xu Chongfeng, Grigorean Gabriela, Ziff Edward B., Neubert Thomas A. (2004). Identification and Verification of Novel Rodent Postsynaptic Density Proteins. Molecular & Cellular Proteomics.

[41] Peng J, Kim MJ, Cheng D, Duong DM, Gygi SP, Sheng M (2004). Semiquantitative proteomic analysis of rat forebrain postsynaptic density fractions by mass spectrometry. J Biol Chem.

[42] Cunha SR, Bhasin N, Mohler PJ (2007). Targeting and stability of Na/ca exchanger 1 in cardiomyocytes requires direct interaction with the membrane adaptor ankyrin-B. J Biol Chem.

[43] Mohler PJ, Davis JQ, Davis LH, Hoffman JA, Michaely P, Bennett V (2004). Inositol 1,4,5-trisphosphate receptor localization and stability in neonatal cardiomyocytes requires interaction with Ankyrin-B. J Biol Chem.

[44] Timmermann DB, Westenbroek RE, Schousboe A, Catterall WA (2002). Distribution of high-voltage-activated calcium channels in cultured γ-aminobutyric acidergic neurons from mouse cerebral cortex. J Neurosci Res.

[45] Zhang Y, Helm JS, Senatore A, Spafford JD, Kaczmarek LK, Jonas EA (2008). PKC-induced intracellular trafficking of Cav2 precedes its rapid recruitment to the plasma membrane. J Neurosci.

[46] Sheng ZH, Rettig J, Takahashi M, Catterall WA (1994). Identification of a syntaxin-binding site on N-type calcium channels. Neuron..

[47] Passafaro M, Rosa P, Sala C, Clementi F, Sher E (1996). N-type ca 2+ channels are present in secretory granules and are transiently translocated to the plasma membrane during regulated exocytosis. J Biol Chem.

[48] Scott MB, Kammermeier PJ (2017). CaV2 channel subtype expression in rat sympathetic neurons is selectively regulated by α2δ subunits. Channels..

